# CD4^+^ T Cell‐Released Extracellular Vesicles Potentiate the Efficacy of the HBsAg Vaccine by Enhancing B Cell Responses

**DOI:** 10.1002/advs.201802219

**Published:** 2019-09-30

**Authors:** Jian Lu, Jing Wu, Feiting Xie, Jie Tian, Xinyi Tang, Hongye Guo, Jie Ma, Ping Xu, Lingxiang Mao, Huaxi Xu, Shengjun Wang

**Affiliations:** ^1^ Department of Laboratory Medicine The Affiliated People's Hospital Jiangsu University Zhenjiang 212002 China; ^2^ Department of Immunology Jiangsu Key Laboratory of Laboratory Medicine School of Medicine Jiangsu University Zhenjiang 212013 China; ^3^ Department of Laboratory Medicine The Fifth People's Hospital of Suzhou Suzhou 215131 China

**Keywords:** B cells, CD4^+^ T cells, CD40L, extracellular vesicles, HBsAg vaccine

## Abstract

T cells secrete bioactive extracellular vesicles (EVs), but the potential biological effects of CD4^+^ T cell EVs are not clear. The main purpose of this study is to investigate the effects of CD4^+^ T cell–derived EVs on B cell responses and examine their role in antigen‐mediated humoral immune responses. In this study, CD4^+^ T cell EVs are purified from activated CD4^+^ T cells in vitro. After immunization with the Hepatitis B surface antigen (HBsAg) vaccine, CD4^+^ T cell EVs‐treated mice show stronger humoral immune responses, which is indicated by a greater Hepatitis B surface antibody (HBsAb) level in serum and a greater proportion of plasma cells in bone marrow. In addition, it is found that EVs released from activated CD4^+^ T cells play an important role in B cell responses in vitro, which significantly promote B cell activation, proliferation, and antibody production. Interestingly, antigen‐specific CD4^+^ T cell EVs are found to be more efficient than control EVs in enhancing B cell responses. Furthermore, it is shown that CD40 ligand (CD40L) is involved in CD4^+^ T cell EVs‐mediated B cell responses. Overall, the results have demonstrated that CD4^+^ T cell EVs enhance B cell responses and serve as a novel immunomodulator to promote antigen‐specific humoral immune responses.

## Graphical abstract

CD4^+^ T cells release bioactive extracellular vesicles. CD4^+^ T cells extracellular vesicles (EVs) can enhance B cell responses by CD40 ligand (CD40L), which significantly promote B cell activation, proliferation, and antibody production in vitro. In addition, the application of CD4^+^ T cell EVs further promotes antigen‐specific humoral immune responses in Hepatitis B surface antigen (HBsAg)‐immunized mice.

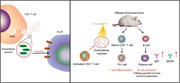

## 1 Introduction

Extracellular vesicles (EVs) are the nanosized spherical membrane particles released from parental cell, which contain mRNA, miRNA, noncoding RNA, protein, lipids, and DNA.^1^, ^2^ EVs can be constitutively or inductively secreted by a variety of cell types, including immune cells, such as dendritic cells (DCs), T cells and B cells.^3^ These spherical membrane vesicles are found in various physiological fluids, such as urine, plasma, cerebrospinal fluid, human milk, and even exudates.^4^, ^5^, ^6^, ^7^, ^8^, ^9^ EVs have been implicated in intercellular interactions, including protein and RNA transfer.^10^, ^11^ They have also been suggested to play a significant role in tumor immunity as both tumor growth promoters and inhibitors.^12^, ^13^

EVs secreted from different T cells, such as CD3^+^ T cells,^14^ CD4^+^ T cells,^15^ and CD8^+^ T cells, have been reported by several groups.^16^ T cell‐released EVs have been suggested to deliver antigen‐specific signals,^15^ atherogenic signals,^17^ and costimulatory signals.^18^ Activated CD4^+^ T cells have been shown to release bioactive EVs expressing CD3, CD4, CD25, T cell receptor (TCR), lymphocyte function associated antigen‐1 (LFA‐1), and Fas ligand (FasL) and to be implicated in immune regulation in DC‐mediated T cell activation.^14^, ^15^ In addition, regulatory T cells (Tregs) are also reported to inhibit the Th1 immune responses by miRNA‐containing EVs.^19^ Moreover, adoptive transferring of autologous Tregs‐released EVs from rats can strengthen kidney function and prolong the survival of kidney allografts posttransplantation.^20^ It seems that T cell‐released EVs play an important role in immune suppression. However, several studies also reported the immune activation of T cell‐released EVs. Wu et al. shown that cytotoxic T cells (CTLs)‐released EVs can enhance the activation of CTLs stimulated by low‐affinity peptides.^21^ It is also reported that T cell‐released EVs are involved in the activation and proliferation of resting CD3^+^ T cells. Together with interleukin‐2, these EVs can induce a relative increase of CD8^+^ T cells.^14^ In summary, these results also revealed the potential of T cell‐released EVs in immune activation. While many studies have focused on immunoregulation of T cell‐released EVs,^22^, ^23^, ^24^ to the best of our knowledge, not many studies have demonstrated the role of CD4^+^ T cell EVs in B cell responses. Interestingly, it seems that activated CD4^+^ T cell EVs are more likely to enhance B cell responses instead of inhibiting it. Therefore, the purpose of our study was to investigate whether EVs released from activated CD4^+^ T cells play a supporting role in B cell responses and can serve as a novel immunomodulator to enhance humoral immune responses.

In this study, CD4^+^ T cell EVs were first shown to be involved in T cell‐mediated B cell responses. Then, we confirmed that CD4^+^ T cell EVs treatment enhances the humoral immune responses in HBsAg‐vaccinated mice. It was shown that CD4^+^ T cell EVs‐treated mice show a greater HBsAb level in serum compared to control mice, which was in an antigen‐dependent manner. We further demonstrated that CD4^+^ T cell EVs promote B cell activation, proliferation, and antibody production in vitro. Interestingly, antigen‐specific CD4^+^ T cell EVs showed a stronger biological function than wild‐type EVs. In addition, using mouse T cell lymphoma EL‐4 cell, we found that CD40L played a significant role in T cell EVs‐mediated B cell responses. Altogether, our study highlighted the B cell stimulatory capacity of EVs released by CD4^+^ T cells. We propose that CD4^+^ T cell EVs could serve as a novel immunomodulator to promote antigen‐specific humoral immune responses in vivo.

## 2 Results

### 2.1 EVs Are Involved in CD4^+^ T Cell‐Mediated B Cell Responses

**Figure 1:**
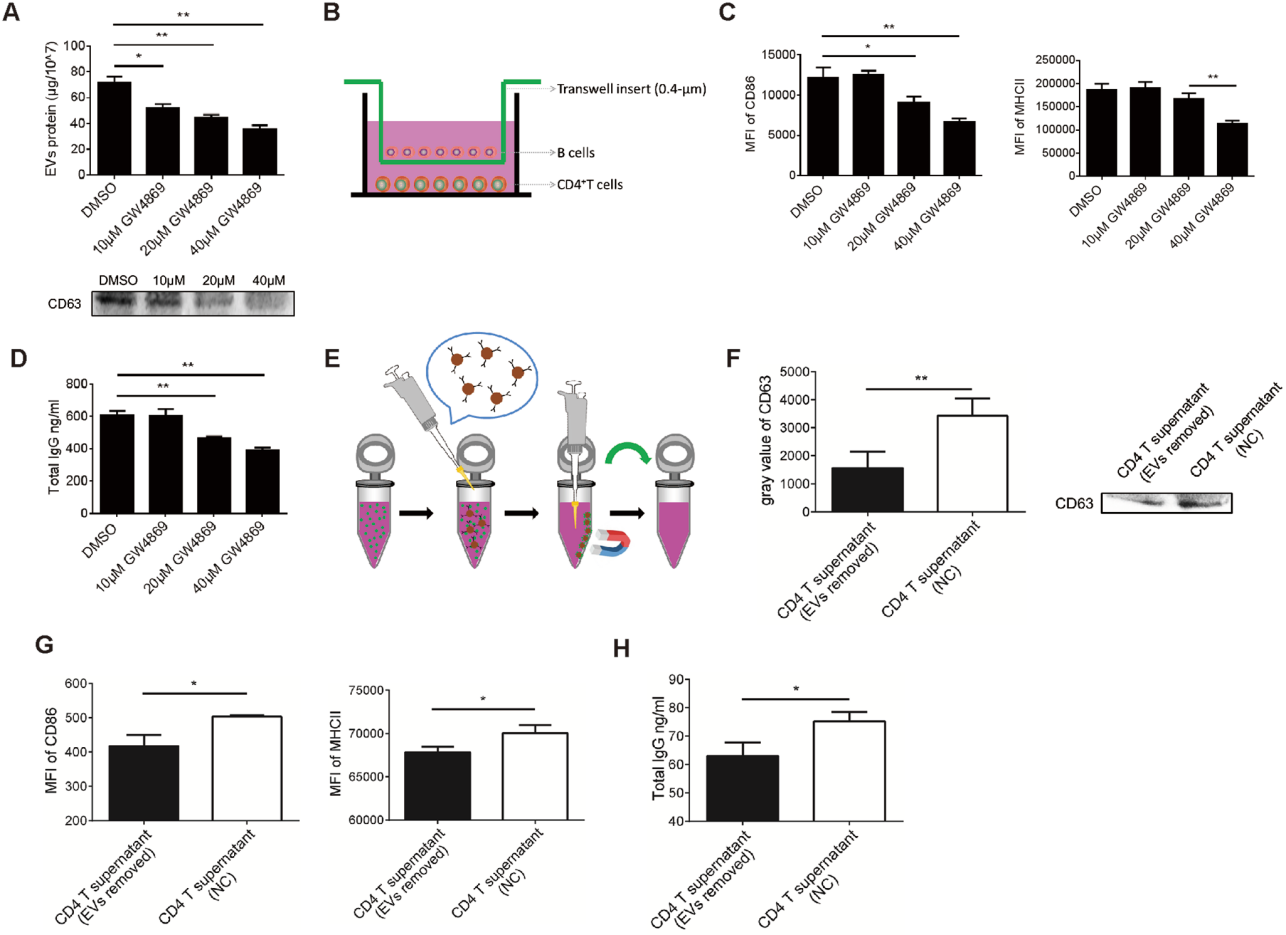
Inhibition of EVs release impairs the function of CD4^+^ T cells in vitro. A) Bicinchoninic acid (BCA) protein assay of total protein (top) and immunoblot analysis of CD63 (bottom) in purified EVs from supernatants of CD4^+^ T cells treated with a control vehicle (dimethyl sulfoxide (DMSO)) or 10, 20, or 40 × 10^−6^ m GW4869 for 48 h. B) A schematic of the Transwell coculture model with B cells in the upper chamber and CD4^+^ T cells in the lower chamber of the well. A porous (0.4 µm) membrane allows the transfer of EVs but precludes direct cell contact. C) Flow cytometry analysis of CD86 and MHCII expression on the surface of B cells. D) Total IgG levels in B cell supernatants were analyzed by enzyme‐linked immunosorbent assay (ELISA). E) A schematic of the utilization of anti‐CD63 microbeads to remove EVs in CD4^+^ T cell supernatant. F) The removal efficiency of CD63‐positive EVs in the supernatant was evaluated by western blotting. G) CD86 and MHCII expression on the surface of B cells cultured with different supernatants for 48 h was analyzed by flow cytometry (FCM). H) B cells were cultured with different supernatants for 72 h, and the total IgG in the supernatant was analyzed by ELISA. *P < 0.05, **P < 0.01, and ***P < 0.001 (Student's *t*‐test). The data are from three independent experiments (A (top), C, D, F (left), G, and H; mean and s.e.m.) or are representative of three independent experiments (A (bottom) and F (right)).

Extracellular vesicles (EVs) have been shown to influence cellular responses by shuttle proteins and RNAs in recipient cells in various settings.^25^ CD4^+^ T cells play an important role in B cell responses in vivo and in vitro. Therefore, we speculated whether EVs released from CD4^+^ T cells are involved in the T‐B cell intercellular interaction. We first used GW4869, an nSMase2 inhibitor,^26^ to inhibit EVs production in vitro. As shown in **Figure** **1**A, GW4869 inhibited EVs production in CD4^+^ T cells in a dose‐dependent manner, while there was no significant effect on the cell viability of CD4^+^ T cells (Figure S1A, Supporting Information). Then, we established an in vitro Transwell coculture system to allow the transfer of EVs but to preclude direct cell contact (Figure 1B) and evaluated the effects of CD4^+^ T cells treated with the EVs inhibitor GW4869 on B cells. It was shown that the reduction in CD86 and major histocompatibility complex class II (MHCII) expression in B cells and the decrease in IgG in the B cell supernatant in the coculture system depended on the extent to which EVs release was inhibited (Figure 1C,D). To exclude the inhibitory effects of GW4869 on B cell responses, B cells were directly cocultured with different concentrations of GW4869, and CD86 and MHCII expression in B cells and IgG production were detected. It was shown that 10, 20, and 40 × 10^−6^ m GW4869 had no significant inhibitory effects on B cells (Figure S1B,C, Supporting Information). In addition, the supernatants of CD4^+^ T cells that were activated in vitro for 48 h were collected, and anti‐CD63 microbeads were used to remove most of the CD63‐positive EVs in supernatants by magnetic sorting (Figure 1E). The removal efficiency was assessed by western blotting. As shown in Figure 1F, compared to the untreated group, the treatment with anti‐CD63 microbeads reduced the content of EVs in the supernatant. Then, we evaluated the effects of EVs in the supernatant on B cell responses in vitro. As shown in Figure 1G, compared to the control supernatant group, the reduction in CD86 and MHCII expression in B cells was observed in the EVs‐removed supernatant group. Moreover, the IgG level in the EVs‐removed supernatant group significantly decreased (Figure 1H). These results showed that CD4^+^ T cells could enhance B cell responses by releasing EVs.

### 2.2 Characterization of EVs from Activated CD4^+^ T Cells

**Figure 2:**
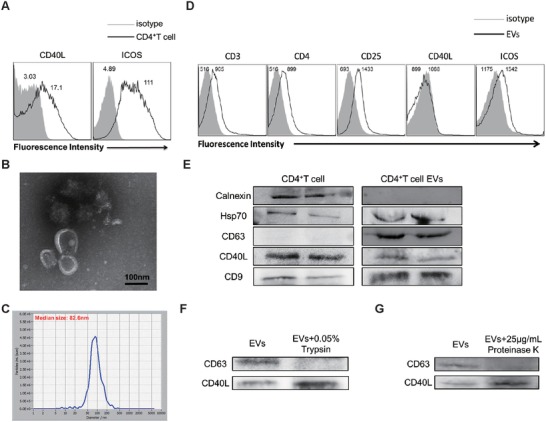
Characterization of EVs released from activated CD4^+^ T cells. A) CD4^+^ T cells from wild‐type mice were activated with plate‐bound anti‐CD3 (2 µg mL^−1^) and soluble CD28 (2 µg mL^−1^) for 24 h, stained with fluorescent conjugated anti‐CD40L and ICOS mAbs or isotype‐matched mAbs and then analyzed by flow cytometry. B) Electron microscopy analysis of CD4^+^ T cell EVs. Representative electron micrographs of EVs are shown. Scale bar: 100 nm. C) Nanoparticle tracking analysis (NTA) of CD4^+^ T cell EVs. D) For the analysis of EVs surface molecules, EVs were first incubated with 2 µL 4 µm diameter aldehyde/sulfate latex beads, then stained with a panel of mAbs (solid lines) or isotype‐matched mAbs (dotted lines) and analyzed by flow cytometry. E) Western blot analysis of CD4^+^ T cell lysates and their EVs using the previously indicated mAbs. 50 µg CD4^+^ T cell EVs were exposed to F) 0.05% trypsin or G) 25 µg mL^−1^ proteinase K, samples were subjected to immunoblot analysis using antibodies against CD63 and CD40L.*P < 0.05 (Student's *t*‐test). The data are representative of three independent experiments (A)–(G).

Upon determining that CD4^+^ T cells enhance B cell responses by releasing EVs, we isolated CD4^+^ T cells from the spleens of wild‐type mice by magnetic cell sorting. After stimulation with anti‐CD3 and CD28 mAbs, CD4^+^ T cells highly expressed CD40L and inducible costimulatory molecule (ICOS) (**Figure** **2**A), which partly indicated its activation state. EVs were prepared in vitro from supernatants of activated CD4^+^ T cells, and their morphology was analyzed by electron microscopy. CD4^+^ T cell EVs showed a typical round or disk‐like morphology (Figure 2B), with a diameter between 50 and 200 nm (Figure 2C). The phenotypes of the EVs were analyzed by flow cytometry. Because of their small size, the EVs were first coated onto 4 µm diameter aldehyde/sulfate latex beads. Similar to their parent CD4^+^ T cells, CD4^+^ T cell EVs expressed CD4^+^ T cell‐related molecules on their surfaces, such as CD4, CD25, and ICOS, but we did not observe the expression of CD40L compared to the expression of isotype control (Figure 2D), which was consistent with a previous study.^27^ In addition, the results of western blot shown that CD4^+^ T cell EVs expressed EVs‐related protein markers, such as heat shock protein 70 (Hsp70), CD63, and CD9. Interestingly, it was shown that CD40L was presented in EVs (Figure 2E), which indicated that CD40L may be expressed within EVs but not on the EVs surface. To further confirm whether EVs contains CD40L, limited trypsin/proteinase K digestion was used to digest proteins not enclosed by lipid membrane.^28^ As shown in Figure 2F,G, trypsin/proteinase K digestion eliminated most of the EVs transmembrane protein CD63 but not CD40L, suggesting that CD40L is contained within EVs. Collectively, these results indicate the successful preparation of CD4^+^ T cell EVs from culture medium.

### 2.3 CD4^+^ T Cell EVs Promote HBsAb Production in HBsAg‐Vaccinated Mice

**Figure 3:**
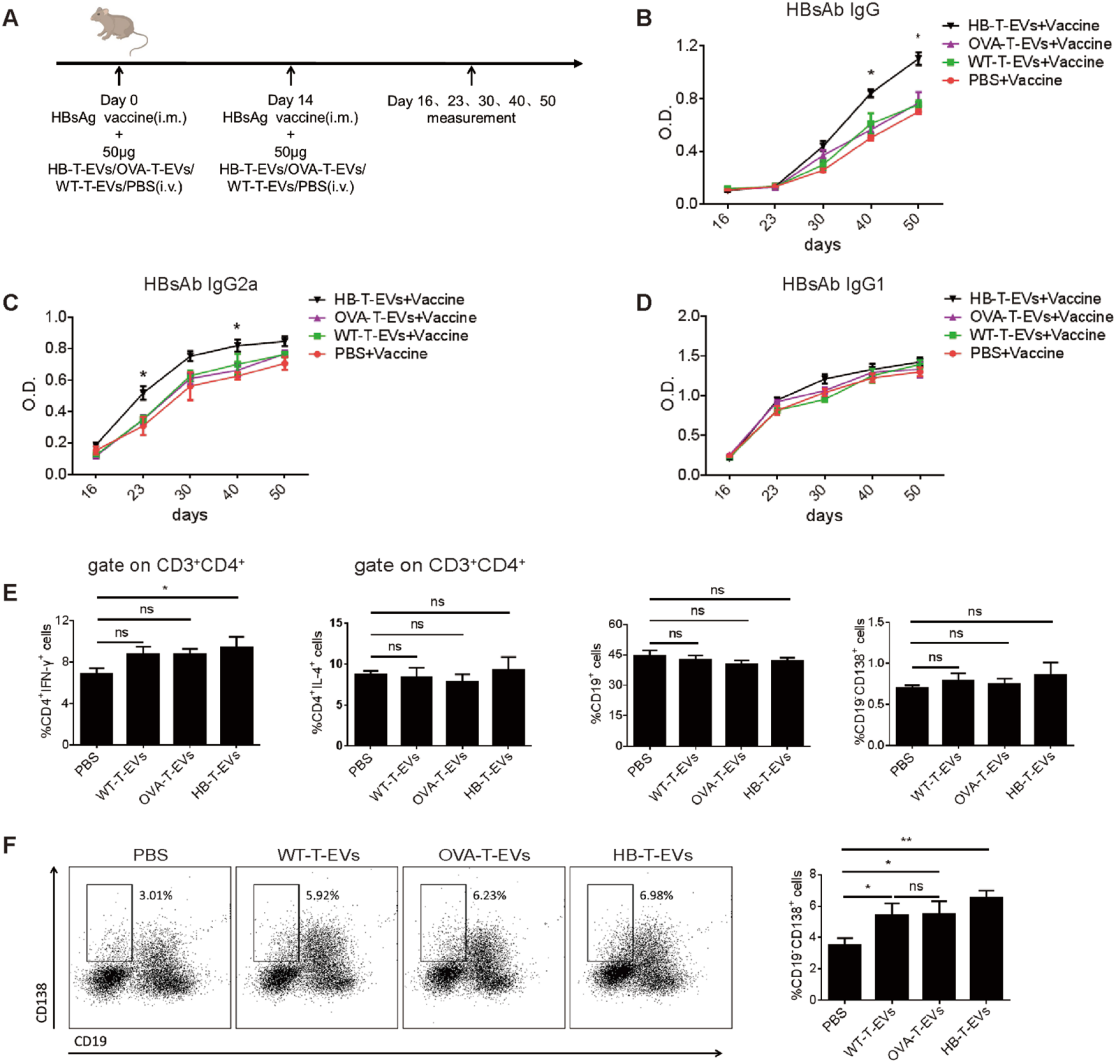
CD4^+^ T cell EVs stimulate the production of HBsAb in HBsAg‐vaccinated mice. A) A schematic of the mouse treatments. The mice were injected with HBsAg vaccine (i.m.) together with HB‐T‐EVs/OVA‐T‐EVs/WT‐T‐EVs or PBS treatment (i.v.), and serum was collected on days 16, 23, 30, 40, and 50. B–D) The absorbance of HBsAb IgG, IgG2a, and IgG1 in serum at different time points of treatment was quantified by ELISA. E) The proportion of spleen Th1 cells, Th2 cells, B cells, and plasma cells were analyzed by flow cytometry at day 50. F) Flow cytometry analysis of bone marrow plasma cells by CD19 and CD138 staining (gate on bone marrow lymphocytes). Representative dot plots of bone marrow cells are shown. *P < 0.05 and **P < 0.01 (Student's *t*‐test). The data are from two independent experiments with five mice per group (B, C, D, E, and F (right); mean and s.e.m.) or are representative of two independent experiments with five mice per group (F (left)).

As a traditional group of helper T cells, CD4^+^ T cells play an important role in B cell activation and humoral immune responses. To investigate whether CD4^+^ T cell EVs promote HBsAb production in HBsAg‐vaccinated mice, we prepared EVs from activated CD4^+^ T cells of HBsAg‐vaccinated mice, Ovalbumin (OVA)‐immunized mice and wild‐type mice (termed HB‐T‐EVs, OVA‐T‐EVs, and WT‐T‐EVs, respectively). The ability of CD4^+^ T cell EVs to stimulate HBsAb production was assessed after two intramuscular (i.m.) injections of HBsAg together with simultaneous intravenous (i.v.) injections of 50 µg HB‐T‐EVs, OVA‐T‐EVs, or WT‐T‐EVs (**Figure** **3**A). As shown in Figure 3B, compared to the phosphate‐buffered saline (PBS‐), OVA‐T‐EVs‐, and WT‐T‐EVs‐treated groups, the level of HBsAb in HBsAg‐vaccinated mice treated with HB‐T‐EVs significantly increased, which was significantly greater than that of the OVA‐T‐EVs‐, WT‐T‐EVs‐, and PBS‐treated groups at day 40 and day 50 (*P* < 0.05). However, the HBsAb level in OVA‐T‐EVs‐ and WT‐T‐EVs‐treated mice was not significantly different from the PBS control group, suggesting that the biological effect of CD4^+^ T cell EVs was antigen‐specific. In addition, we also showed that antigen‐specific CD4^+^ T cell EVs can stimulate sheep red blood cell (SRBC)‐specific IgG production in BALB/C mice immunized with SRBC, which was in agreement with the abovementioned results (Figure S2, Supporting Information). To further investigate the effect of CD4^+^ T cell EVs on the production of HBsAb subtypes, we analyzed serum HBsAb IgG2a and IgG1 levels using enzyme‐linked immunosorbent assay (ELISA), which reflected the Th1 and Th2 responses, respectively.^29^, ^30^ By analyzing the HBsAb subtypes, we found that HB‐T‐EVs mainly enhanced the production of HBsAb IgG2a (Figure 3C) but have no effect on HBsAb IgG1 production (Figure 3D). Therefore, the enhancement of the antibody response mediated by CD4^+^ T cell EVs was mainly attributed to the increase in Th1 antibodies. In addition, flow cytometry analysis showed that CD4^+^ T cell EVs increased the proportion of Th1 cells in the spleen, while have no significant effect on Th2 cells, B cells and plasma cells (Figure 3E). Interestingly, the proportion of bone marrow plasma cells was greater in CD4^+^ T cell EVs‐treated groups than that in the control group (Figure 3F). Overall, our data demonstrated that CD4^+^ T cell EVs stimulated HBsAb production in HBsAg‐vaccinated mice in an antigen‐dependent manner, primarily by increasing Th1 type antibody production. In addition, CD4^+^ T cell EVs can also increase the proportion of spleen Th1 cells and bone marrow plasma cells in an antigen‐independent manner.

### 2.4 CD4^+^ T Cell EVs Bind to B Cells and Are Taken Up by B Cells

**Figure 4:**
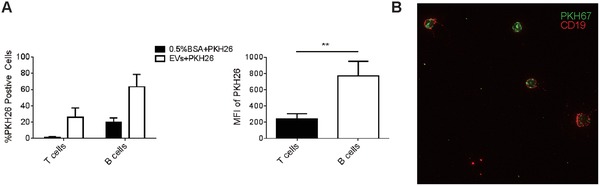
CD4^+^ T cell EVs bind to B cells and are taken up by B cells. A) A total of 5 × 10^5^ spleen cells from OVA‐immunized mice were incubated in 50 µg PKH26‐labeled OVA‐T‐EVs for 4 h at 37 °C (5% CO_2_). The cells were then stained with fluorescent conjugated anti‐CD3 and CD19 mAbs for 30 min. The percentage of PKH26‐positive cells and the mean fluorescence intensity (MFI) of PKH26 in T and B cells were analyzed by flow cytometry. B) Confocal microscopy analysis of CD19^+^ B cells (red) incubated with PKH67‐labeled OVA‐T‐EVs (green) for 4 h at 37 °C. **P < 0.01 (Student's *t*‐test). The data are from three independent experiments (A; mean and s.e.m.) or are representative of three independent experiments (B).

To further investigate the mechanism of CD4^+^ T cell EVs‐mediated HBsAb responses, we assessed whether CD4^+^ T cell EVs could bind to T and B cells in vitro. Therefore, spleen cells from OVA‐immunized mice were cocultured with PKH26‐labeled OVA‐T‐EVs or WT‐T‐EVs for 4 h and then analyzed by flow cytometry. As shown in **Figure** **4**A, ≈60% of CD19^+^ B cells in the OVA‐T‐EVs cocultured group expressed PKH26, which was greater than CD3^+^ T cells (≈25%). The mean fluorescence intensity of PKH26 on the surface of B cells was almost threefold greater than that on T cells, indicating that B cells can bind more CD4^+^ T cell EVs than T cells. The results were nearly identical in the WT‐T‐EVs cocultured group (data not shown). Upon determining that CD4^+^ T cell EVs can bind to B cells, we next investigated whether the EVs were also taken up by B cells. To analyze the uptake of EVs, OVA‐T‐EVs and WT‐T‐EVs were labeled with the lipid dye PKH67 and cocultured with B cells for 4 h at 37 °C. Confocal laser scanning microscopy (CLSM) analysis showed the intracellular localization of CD4^+^ T cell EVs in B cells in both the OVA‐T‐EVs (Figure 4B) and WT‐T‐EVs (data not shown) cocultured groups. Together, these findings indicate that CD4^+^ T cell EVs bind to B cells and are taken up by B cells.

### 2.5 CD4^+^ T Cell EVs Play an Important Role in B Cell Responses In Vitro

**Figure 5:**
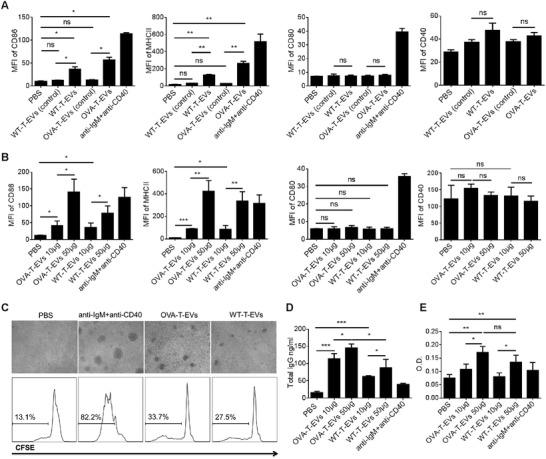
CD4^+^ T cell EVs promote B cell activation, proliferation and antibody production in vitro. A) A total of 5 × 10^5^ B cells isolated from OVA‐immunized mice were incubated with 50 µg WT‐T‐EVs (control)/WT‐T‐EVs or OVA‐T‐EVs (control)/OVA‐T‐EVs at 37 °C for 48 h and then incubated with fluorescent conjugated anti‐CD86, CD80, CD40, and MHCII mAbs. The data were analyzed by FCM. B) B cells isolated from OVA‐immunized mice were incubated with different doses of OVA‐T‐EVs or WT‐T‐EVs for 48 h. The activation of B cells was evaluated by the expression of CD86, CD80, CD40, and MHCII. C) A total of 5 × 10^5^ CFSE‐labeled B cells were incubated with 50 µg OVA‐T‐EVs or WT‐T‐EVs for 4 d, and the proliferation of B cells was analyzed by microscopy and flow cytometry. Furthermore, the culture supernatant was collected, and the total D) IgG and E) OVA‐specific IgG antibodies were analyzed by ELISA. *P < 0.05, **P < 0.01, and ***P < 0.001 (Student's *t*‐test). The data are from three independent experiments (A, B, D, and E; mean and s.e.m.) or are representative of three independent experiments (C).

To further verify the effects of these EVs on B cell responses, B cells from OVA‐immunized mice were incubated for 48 h with CD4^+^ T cell EVs and subsequently stained with fluorescent conjugated anti‐CD86, CD80, CD40, and MHCII mAbs to investigate their activation state. First, we wondered whether only CD3/CD28‐stimulated CD4^+^ T cell derived EVs have the biological functions, and EVs from CD3/CD28‐unstimulated CD4^+^ T cells from wild‐type mice and OVA‐immunized mice were used (termed WT‐T‐EVs (control) and OVA‐T‐EVs (control), respectively). As shown in **Figure** **5**A, high expression of CD86 and MHCII on the surface of B cells was observed in both WT‐T‐EVs and OVA‐T‐EVs treated groups but not in the WT‐T‐EVs (control) and OVA‐T‐EVs (control) treated groups, indicating that only highly activated CD4^+^ T cell‐released EVs were bioactive and could induce B cell activation. However, the EVs treatment did not affect the expression of CD80 and CD40 on the surface of B cells. We further demonstrated that CD4^+^ T cell EVs promote B cell activation in a dose‐dependent manner. As shown in Figure 5B, significant expression of CD86 or MHCII was observed on the surface of B cells when 10 µg WT‐T‐EVs or OVA‐T‐EVs was added, and a trend toward increased expression with 50 µg EVs was observed. Moreover, OVA‐T‐EVs showed a much stronger biological effect than WT‐T‐EVs. To further investigate whether CD4^+^ T cell EVs induce the proliferation of B cells, B cells isolated from OVA‐immunized mice were labeled with carboxyfluorescein diacetate succinimidyl ester (CFSE), and cell proliferation was assessed by flow cytometry 4 d after stimulation with 50 µg CD4^+^ T cell EVs. As shown in Figure 5C, compared to the PBS‐treated group (13.1%), the WT‐T‐EVs treatment induced a significant proliferation rate (27.5%) of B cells, and the OVA‐T‐EVs treatment induced an even greater proliferation rate (33.7%). Antibody secretion can reflect the function of B cells. Therefore, we analyzed the total IgG and OVA‐specific IgG level in B cell supernatant. As shown in Figure 5D,E, both WT‐T‐EVs‐ and OVA‐T‐EVs‐treated B cells secreted a greater amount of total IgG and OVA‐specific IgG than the PBS‐treated group in a dose‐dependent manner. Moreover, OVA‐T‐EVs‐treated B cells secreted a much greater amount of total IgG and OVA‐specific IgG than WT‐T‐EVs‐treated cells. These results showed that CD4^+^ T cell EVs played an important role in B cell activation, proliferation and antibody production in vitro. Taken together, these data indicate that antigen‐specific CD4^+^ T cell EVs possess a stronger biological function than wild‐type EVs.

### 2.6 CD40L Is Involved in T Cell EVs‐Mediated B Cell Responses In Vitro

**Figure 6:**
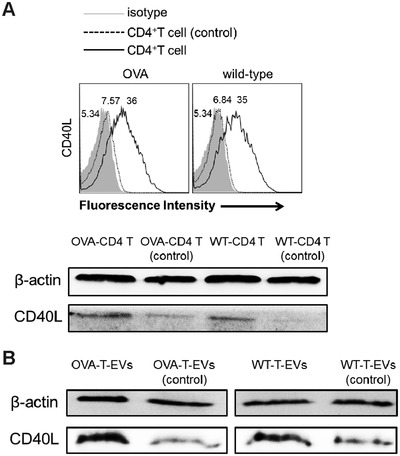
Activated CD4^+^ T cell EVs highly express CD40L. A) CD3/CD28‐stimulated (black solid lines) or control (black dotted lines) CD4^+^ T cells from OVA‐immunized mice and wild‐type mice were stained with a fluorescent conjugated anti‐CD40L mAb and then analyzed by flow cytometry (top). Total CD40L expression in OVA‐CD4 T/WT‐CD4 T and OVA‐CD4 T (control)/WT‐CD4 T (control) were compared by western blotting (bottom). B) The expression of CD40L in OVA‐T‐EVs/WT‐T‐EVs and OVA‐T‐EVs (control)/WT‐T‐EVs (control) were compared by western blotting. One representative experiment of three experiments is shown.

CD40L is a type II transmembrane cytokine expressed on activated CD4^+^ T cells upon TCR stimulation. It engages CD40 on the surface of B cells for T cell‐dependent antibody responses.^31^ CD40L is essential for the development and maintenance of the germinal center and the processes of B cell affinity maturation, immunoglobulin class switching, and long‐lived plasma cell generation.^32^, ^33^ Using flow cytometry, high expression of CD40L was observed on CD3/CD28‐stimulated CD4^+^ T cells from OVA‐immunized and wild‐type mice (termed OVA‐CD4 T and WT‐CD4 T, respectively), while there was quite low CD40L expression on CD3/CD28‐unstimulated CD4^+^ T cells from OVA‐immunized and wild‐type mice (termed OVA‐CD4 T (control) and WT‐CD4 T (control), respectively). However, there was no significant difference between OVA‐CD4 T and WT‐CD4 T in CD40L expression. In addition, total CD40L protein expression level in CD4^+^ T cells were also analyzed by western blotting, and the results were consistent with those observed using flow cytometry (**Figure** **6**A). We further compared the difference in CD40L expression between OVA‐T‐EVs/WT‐T‐EVs and OVA‐T‐EVs (control)/WT‐T‐EVs (control) by western blotting. As shown in Figure 6B, OVA‐T‐EVs and WT‐T‐EVs had greater amounts of CD40L than control EVs. Therefore, based on the differential expression of CD40L both on CD3/CD28‐stimulated and control CD4^+^ T cells and their released EVs, we hypothesize that CD40L may play a critical role in CD^+^ T cell EV‐mediated B cell responses in vitro.

**Figure 7:**
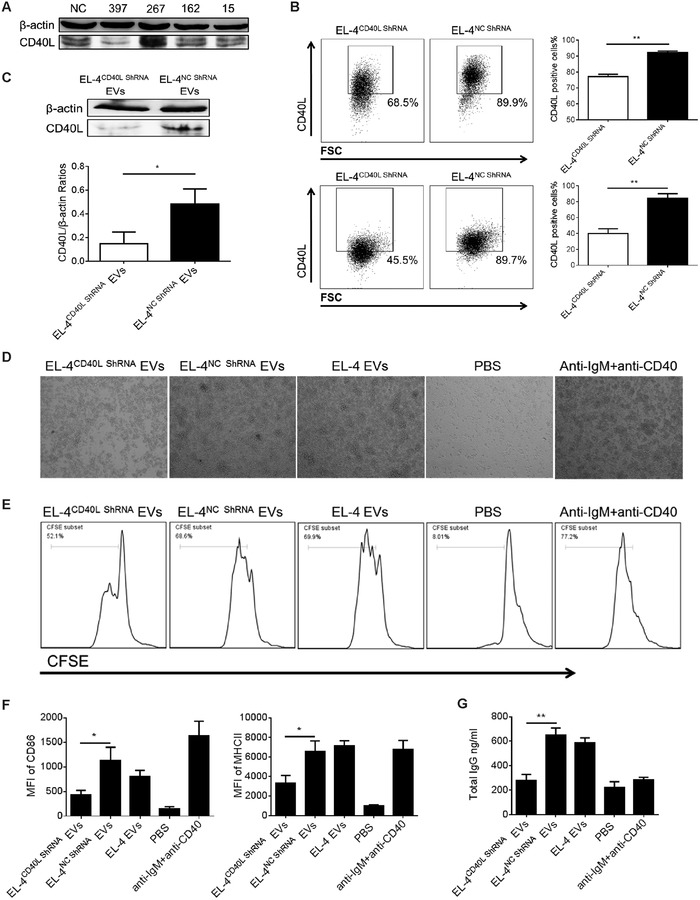
CD40L is involved in T cell EVs‐mediated B cell responses in vitro. A) Different CD40L shRNA recombinant plasmids were transfected into EL‐4 cells using Lipofectamine 2000 transfection reagent, positive clones were screened by G418, CD40L interference efficiency was analyzed by western blotting, and the most efficient plasmid was filtered out. B) Surface (top) and total CD40L (bottom) expression of EL‐4^CD40L shRNA^ and EL‐4^NC shRNA^ cells were analyzed by FCM. C) CD40L expression of EVs released from EL‐4^CD40L shRNA^ and EL‐4^NC shRNA^ cells were analyzed by western blotting. The interference efficiency was indicated by the gray level ratio of CD40L/β‐actin. A total of 5 × 10^5^ CFSE‐labeled B cells isolated from wild‐type mice were incubated with 50 µg EL‐4^CD40L shRNA^ EVs or EL‐4^NC shRNA^ EVs for 72 h. The proliferation of B cells was evaluated by D) microscopy and E) FCM. For the analysis of B cell activation, 5 × 10^5^ B cells were incubated with 50 µg EL‐4^CD40L shRNA^ EVs or EL‐4^NC shRNA^ EVs for 48 h; the expression of CD86 and MHCII was analyzed by F) FCM, and G) the total IgG in culture supernatant was analyzed by ELISA. *P < 0.05 and **P < 0.01 (Student's *t*‐test). The data are from three independent experiments (B (right), F, and G; mean and s.e.m.) or are representative of three independent experiments (A, B (left), C(top), D, and E).

We have shown that activated CD4^+^ T cells highly express CD40L in vitro, and their EVs are involved in B cell responses, which contain CD40L molecules. Interestingly, Gardell and Parker found that CD40L can be transferred from T cells to B cells, which is correlated with B cell activation.^34^ EL‐4 cells as a class of murine T‐lymphoma cell line, it has been reported to strongly stimulate B cells, which naturally express CD40L, to proliferate and differentiate in vitro and is a good model for studying the intercellular interaction between T cells and B cells in vitro.^35^, ^36^ We demonstrated that EVs released from EL‐4 cells shared the same ability as CD4^+^ T cell EVs, which highly express CD40L and can promote B cell activation, proliferation, and antibody production in vitro (Figure S3, Supporting Information). To further investigate whether CD40L is involved in CD4^+^ T cell EVs‐mediated B cell responses, we constructed a plasmid encoding CD40L shRNA and transfected it into EL‐4 cells to establish a CD40L knockdown cell line. As shown in **Figure** **7**A, EL‐4 cells transfected with recombinant plasmid 397 showed the lowest CD40L expression compared to the other plasmids and control plasmids. Using this CD40L knockdown EL‐4 cell line, we further verified both cell surface and intracellular CD40L expression. As shown in Figure 7B, CD40L shRNA‐transfected EL‐4 cells (EL‐4^CD40L shRNA^) showed lower surface CD40L expression (68.5%) than control EL‐4 cells (EL‐4^NC shRNA^) (89.9%). We also analyzed the cell surface and intracellular CD40L expression simultaneously, which indicated the total CD40L expression in EL‐4 cells. The total CD40L expression in EL‐4^CD40L shRNA^ cells was much less than that in EL‐4^NC shRNA^ cells, which was decreased by almost half. Moreover, the EVs released from EL‐4^CD40L shRNA^ cells also had a lower CD40L expression level than control EVs (Figure 7C). To further evaluate the functional difference between EL‐4^CD40L shRNA^ EVs and EL‐4^NC shRNA^ EVs, B cells isolated from BALB/C mice were labeled with CFSE, and proliferation was analyzed using flow cytometry 3 d after stimulation with 50 µg EVs. As shown in Figure 7D, clump formation was easily observed in the EL‐4^NC shRNA^ EVs‐, EL‐4 EVs‐treated, and EL‐4‐positive control groups, while it was difficult to find any clump formation in the EL‐4^CD40L shRNA^ EVs‐treated group. Flow cytometry analysis also showed that the EL‐4^CD40L shRNA^ EVs‐treated group had a lower percentage of CFSE‐positive cells (52.1%) than the EL‐4^NC shRNA^ EVs‐ and EL‐4 EVs‐treated groups (68.6% and 69.9%, respectively) (Figure 7E). In addition, compared to EL‐4^NC shRNA^ EVs and EL‐4 EVs, the ability of EL‐4^CD40L shRNA^ EVs to promote CD86 and MHCII expression on the surface of B cells was reduced (Figure 7F). Moreover, total IgG in the supernatant of B cells treated with EL‐4^CD40L shRNA^ EVs was much less than that treated with EL‐4^NC shRNA^ EVs. Overall, using the EL‐4 cell line, our results demonstrate that the CD40L molecule is involved in T cell EVs‐mediated activation, proliferation and antibody production of B cells.

## 3 Discussion

In this study, we provide evidence that CD4^+^ T cell EVs can be taken up by B cells and thereby influence B cell biology. We hypothesize that CD4^+^ T cell EVs can serve as an immunomodulator to enhance HBsAb responses in HBsAg‐vaccinated mice. CD4^+^ T cells isolated from HBsAg‐vaccinated mice, OVA‐immunized mice and wild‐type mice were used as constitutive sources for EVs. Our data demonstrate that only antigen‐specific HB‐T‐EVs significantly increase the HBsAb level in mouse serum, primarily by increasing the production of IgG2a, which is a Th1 antibody type. Interestingly, although there was no significant difference of serum HBsAb level between OVA‐T‐EVs/WT‐T‐EVs‐treated group and PBS‐treated group, the proportion of bone marrow plasma cell in OVA‐T‐EVs/WT‐T‐EVs‐treated group was still higher than that in PBS‐treated group. CD4^+^ T cell EVs as a foreign substance, it may carry several functional molecules and contribute to the induction of total plasma cell, but it may have no effect on the antigen‐specific plasma cell for the lack of antigen‐specific signal. EVs originate from internal budding of the plasma membrane during endocytic internalization, they are contained within multivesicular bodies (MVBs) and have incorporated recycled proteins from coated pits in the cellular membrane. The MVBs can further fuse with the plasma membrane to release EVs through exocytosis.^37^ Therefore, antigen‐specific T cell‐released EVs can get antigen‐specific TCR molecule by this way. It is known to all that adaptive immune responses are mediated by antigen‐specific signal, and T cell‐released EVs have been reported to express antigen‐specific TCR molecules.^15^, ^38^ Therefore, HB‐T‐EVs may express the HBsAg‐specific TCR, and thus enhance the HBsAb responses.

Although EVs samples were prepared from the supernatant by differential centrifugation and ultrafiltration membrane technology followed by the use of an EVs extraction kit, which already reduced the protein contamination as much as possible. The purity of our EVs samples were still less than ultracentrifugation. Based on the limitation of this method, EVs specific removed supernatant model was designed, we used CD63 microbeads by magnetic cell sorting to remove the most EVs in supernatant to evaluate its effects on B cell responses. Using this method, we think it will be more convincing that EVs play an important role in CD4^+^ T cell‐mediated B cell responses in vitro. However, it is still necessary for us to improve the EVs purity for the further studies.

We have demonstrated that CD4^+^ T cell EVs play an important role in B cell responses in vitro. It was shown that CD4^+^ T cell EVs can significantly promote the activation and antibody production of B cells. Besides, it can also dose‐dependently promote the CD86 and MHCII expression on B cells. However, it was also reported that MHCII molecule is expressed in T cell EVs,^27^ and MHCII/peptide complexes can be even transferred from T cells to B cells by EVs.^39^ Therefore, our study cannot rule out the possibility that the increase of MHCII on B cells is mediated by the transferred MHCII from CD4^+^ T cell EVs. In vivo, the mechanism by which EVs communicate with the adaptive immune compartment in HBsAg‐vaccinated mice remains unclear. Furthermore, our study did not address whether the EVs signal is delivered directly to B cells or indirectly through other cell types, such as monocytes and DCs, which are also involved in B cell responses.^40^, ^41^ Therefore, it is quite important to confirm the target cells that preferentially take up EVs, and further studies are still needed to clearly define the signaling mechanism. Interestingly, it was reported that CD4^+^ T cell EVs highly expressed TCR and FasL, and CD4^+^ T cell EVs‐treated DCs can silence the T cell responses in antigen‐specific way, which indicated that CD4^+^ T cells can also suppress immune responses. This may be associated with the downregulation or masking of the MHCII on DCs by CD4^+^ T cell EVs and induction of apoptosis in Fas expressing DCs via the Fas/FasL pathway.^15^ In this study, DCs were first treated with CD4^+^ T cell EVs, then these immunoregulatory DCs were used to treat tumor‐bearing mice. By contrast, our study primarily focused on the effects of CD4^+^ T cell EVs itself and evaluated its effects on antigen‐mediated humoral immune responses, the animal model and experimental purpose are different. Almost all living cells can secrete EVs, a large number of studies indicated that the functions of EVs seem to be similar to their sources. Although several studies indicated that T cell EVs shown the immunosuppressive properties, which seem to be inconsistent with our conclusions. It may be associated with the different experimental designs and the different target cells. Therefore, further studies are still needed to clearly define the functions of CD4^+^ T cell EVs.

Using the EL‐4 cell line, we showed that CD40L is involved in T cell EVs‐mediated B cell responses in vitro (Figure 7). Interestingly, Sprague et al. have previously shown that platelet‐derived membrane vesicles are sufficient to deliver CD40L to stimulate antigen‐specific IgG production and modulate germinal center formation,^42^ which is consistent with our results. Although it has been known for decades that the CD40L‐CD40 interaction is key to T cell‐dependent antibody responses and plays an important role in the proliferation and differentiation of B cells, the molecular mechanism involved in CD40L signaling remains unclear. Recently, Gardell and Parker speculated that CD40L may be released in extracellular vesicles, which can be further taken up by B cells, thus activating the B cells.^34^, ^43^ The release of full‐length CD40L in membrane vesicles by platelets and mast cells has been reported, which is involved in B cell activation and antibody production.^42^, ^44^ In this study, our data first showed that CD4^+^ T cell EVs contain CD40L molecules, but we did not observe any positive expression of CD40L on the EVs surface. Based on the results of limited trypsin/proteinase K digestion of EVs transmembrane proteins, we speculated that CD40L is contained within the CD4^+^ T cell EVs. Interestingly, the CD40L‐containing EVs were shown to activate B cells in vitro (Figures 6 and 7), indicating that the process of EVs‐mediated B cell responses may be independent of the CD40L‐CD40 direct interaction on the membrane surface. It has been reported that EVs can be taken up by target cells via various routes, such as direct membrane fusion, phagocytosis, endocytosis, and even pinocytosis.^45^ In addition, studies have also shown that these nanovesicles can communicate with target cells by releasing their segregated agents shortly upon discharge. Upon release, EVs do not remain intact and breakdown and then release their contents into the extracellular space,^46^, ^47^ thus exerting their biological effects. This may partly explain the mechanism of CD40L‐containing EVs in B cell responses. Moreover, transforming growth factor‐β and interleukin‐10 containing EVs have already been shown to inhibit CD4^+^ T cell proliferation and induce regulatory T cells, which may depend on the abovementioned mechanism.^48^, ^49^ In our study, we proposed that CD40L can be partially discharged from CD4^+^ T cell EVs and combine with CD40 on the B cell surface, thus enhancing the B cell responses. However, there must be other mechanisms of CD40L‐containing EVs in B cell responses. Intercellular communication associated with the trafficking and release of CD40L from EVs needs to be further defined.

CD40L plays a critical role in the generation of primary effector T cell responses in acute viral infection.^50^ CD8^+^ T cells can be accompanied by CD4^+^ T cells through CD40L/CD40 interactions, which is fundamental for CD8^+^ T cell memory generation.^51^ Harcourt et al. have previously shown that CD40L improved the durability of respiratory syncytial virus DNA vaccination in BALB/C mice.^52^ Interestingly, our results showed that CD40L‐positive CD4^+^ T cell EVs‐treated HBsAg‐vaccinated mice had a greater proportion of Th1 cells compared to PBS‐treated mice. Moreover, we also showed that these CD40L‐positive CD4^+^ T cell EVs promote the proliferation of CD8^+^ T cells in vitro (Figure S4, Supporting Information). Therefore, CD4^+^ T cell EVs may have a dual function both in humoral and cellular immune responses, which indicates its tremendous application potential.

In summary, our data indicate that CD4^+^ T cell EVs promote HBsAb production in HBsAg‐vaccinated mice in an antigen‐dependent manner. The in vitro experiments show that CD4^+^ T cell EVs significantly promote B cell activation, proliferation, and antibody production. We further demonstrate that CD40L is involved in T cell EVs‐mediated B cell responses. Together, we provide new insights into B cell responses mediated by CD4^+^ T cell EVs and offer new directions for the research and development of a novel immunomodulator.

## 4 Experimental Section

*Antibodies and Reagents*: Phycoerythrin (PE)‐conjugated anti‐CD40L, CD25, CD3, CD86, CD80, CD40, MHCII, IL‐4, and IFN‐γ mAbs, fluorescein isothiocyanate‐conjugated anti‐CD4, CD8, and CD19 mAbs, PE‐Cy5‐conjugated anti‐CD4 and ICOS mAbs, and functional grade purified anti‐CD3, CD28, IgM, and CD40 mAbs were all obtained from eBioscience (San Diego, CA, USA). A CD4^+^ T cell isolation kit, antibiotin microbeads, streptavidin PE, and biotin‐labeled mAbs specific for CD19, CD138, and CXCR5 were obtained from Miltenyi Biotec (Bergisch Gladbach, Germany). Anti‐CD9, CD40L, Calnexin, Hsp70, and CD63 mAbs were obtained from Abcam (Cambridge, UK). An anti‐β‐actin mAb and HRP‐conjugated secondary Abs were obtained from Cell Signaling Technology (MI, CA). ExosomeQuick‐TCTM and ExoELISA‐ULTRA CD63 kits were obtained from SBI (Mountain View, CA, USA). A MicroBCA protein assay kit was obtained from Beijing ComWin Biotech (Beijing, China). A mouse total IgG ELISA kit was obtained from MultiSciences (Hangzhou, China). The HBsAg vaccine was obtained from Jintan Biotechnology Co., Ltd. (Shijiazhuang, China). Ovalbumin (OVA), complete Freund's adjuvant (CFA), GW4869, PKH67 green fluorescent cell linker kit and PKH26 red fluorescent cell linker kit were obtained from Sigma‐Aldrich (St. Louis, MO, USA). A CellTrace CFSE cell proliferation kit and exosome CD63 isolation/detection reagent were obtained from Invitrogen (Carlsbad, CA, USA).

*Mice and Cell Lines*: Female BALB/C mice (6–8 weeks old) were purchased from the Animal Research Center of Jiangsu University (Zhenjiang, China) and housed in a specific pathogen‐free facility. The experimental protocols were approved by the Jiangsu University Animal Ethics and Experimentation Committee. Mouse T cell lymphoma EL‐4 cells were obtained from the Cell Bank of the Chinese Academy of Sciences (Shanghai, China).

*CD4^+^ T Cell Isolation*: CD4^+^ T cells were isolated from splenocytes of BALB/C mice with a CD4^+^ T cell positive isolation kit as previously described.^53^ The purity was assessed by measuring the expression of CD3 and CD4 using flow cytometry (FCM), and the purity was >95%. The CD4^+^ T cells were activated in vitro with plate‐bound 2 µg mL^−1^ anti‐CD3 mAb and soluble 2 µg mL^−1^ CD28 mAb.

*EVs Isolation and Analysis*: Isolated CD4^+^ T cells (1.5 × 10^6^ cells mL^−1^) were cultivated in a 24‐well plate and stimulated with plate‐bound anti‐CD3 mAb (5 µg mL^−1^) and soluble CD28 mAb (2 µg mL^−1^) at 37 °C and 5% CO_2_ for 24 h in T cell conditioned medium (RPMI 1640 with 10% fetal bovine serum that had been ultracentrifuged at 100 000 × *g* for 16 h at 4 °C). The culture supernatant of CD4^+^ T cells was harvested. EVs were purified from the supernatant by differential centrifugation and ultrafiltration membrane technology followed by the use of an EVs extraction kit, as previously described.^11^ Briefly, cells and cellular debris were removed by sequential centrifugation at 300 × *g* for 20 min, 1000 × *g* for 30 min, and 10 000 × *g* for 30 min. The supernatant was passed through a 0.22 µm microcentrifuge filter and acquired by an ultrafiltration membrane with a molecular weight cut‐off ranging from 2 to 100 kDa. The filtrate was completely mixed with EVs isolation reagent (v/v = 5:1) and incubated for 16 h at 4 °C. Finally, the mixture was centrifuged at 1500 × *g* for 30 min, and the precipitate consisted of CD4^+^ T cell EVs. The EVs were dissolved in PBS and stored at −80 °C. The protein concentration of CD4^+^ T cell EVs was quantified using a MicroBCA protein assay kit, and EVs abundance was quantified using an ExoELISA‐ULTRA CD63 kit according to the manufacturer's instructions. EVs derived from CD4^+^ T cells of HBsAg‐vaccinated mice, OVA‐immunized mice and wild‐type mice were termed HB‐T‐EVs, OVA‐T‐EVs, and WT‐T‐EVs, respectively. To prepare EL‐4 EVs, EL‐4 cells were cultivated in a cell culture flask at 37 °C and 5% CO_2_ for 24 h in T cell conditioned medium (RPMI1640 with 10% fetal bovine serum that had been ultracentrifuged at 100 000 × *g* for 16 h at 4 °C). The culture supernatant of EL‐4 cells was harvested. The steps of EVs preparation and protein quantification were the same as those mentioned for CD4^+^ T cell EVs. The EVs derived from EL‐4 cells were termed EL‐4 EVs, and EVs derived from EL‐4 cells transfected with CD40L shRNA or NC shRNA was termed EL‐4^CD40L shRNA^ EVs and EL‐4^NC shRNA^ EVs, respectively.

*Electron Microscopy*: For electron microscopy analysis, CD4^+^ T cell EVs were fixed in 4% paraformaldehyde at 4 °C for 1 h. Then, the cell pellets were placed on a formvar‐coated grid and negatively stained with 3% (w/v) aqueous phosphotungstic acid. Sections were observed using transmission electron microscopy (Tecnai‐12, Philips, Amsterdam, Netherlands).

*Nanoparticle Tracking Analysis of EVs*: Particle sizes of CD4^+^ T cell EVs and EL‐4 EVs were analyzed by nanoparticle tracking analysis using a ZetaView PMX 110 (Particle Metrix, Meerbusch, Germany) and the corresponding software ZetaView 8.04.02.

*Flow Cytometry Analysis*: For cell surface staining, single‐cell suspensions were collected and stained with corresponding antibodies for 30 min at 4 °C. For intracellular staining of Th1 and Th2, single‐cell suspensions were first stimulated with 50 ng mL^−1^ phorbol myristate acetate), 1 µg mL^−1^ ionomycin, and 2 µg mL^−1^ monensin. After 5 h, the cells were stained with fluorescent conjugated anti‐CD3 and anti‐CD4 mAbs, fixed, permeabilized, and stained with fluorescent conjugated anti‐IL‐4 or anti‐IFN‐γ mAb as previously described.^54^ For staining of total CD40L in EL‐4 cells, single‐cell suspensions were first stained with fluorescent conjugated anti‐CD40L mAb, then fixed, permeabilized, and stained with fluorescent conjugated anti‐CD40L mAb again according to the intracellular staining kit instructions. For EVs analysis, 200 µg CD4^+^ T cell EVs in PBS were incubated with 1 µL aldehyde/sulfate latex beads (4 µm diameter) for 15 min at room temperature, with a final volume of 20 µL. The mixture was then transferred to 500 µL PBS with gentle shaking for 30 min. The reaction was stopped with 100 × 10^−6^ m glycine and 2% BSA in PBS and rotated at room temperature for 30 min. EVs‐coated beads were washed twice in 2% BSA/PBS, centrifuged for 3 min at 4000 rpm, then blocked with 10% BSA/PBS with gentle shaking for 30 min at room temperature and washed twice. Next, the EVs‐coated beads were incubated with corresponding fluorescent conjugated mAbs with gentle shaking for 1 h at 4 °C and washed twice. Finally, the beads were analyzed using an FACSCalibur flow cytometer (Becton Dickinson, Mountain View, CA) and FlowJo software.

*Western Blot Analysis*: 50 mg of EVs or crude proteins extracted from cell lysates were separated by 12% SDS‐PAGE and transferred onto Immobilon polyvinylidene membranes (Bio‐Rad, Hercules, CA, USA). The membranes were blocked with 5% BSA in TBST for 1 h at room temperature and then incubated with the corresponding primary Abs overnight at 4 °C. After incubating with HRP‐conjugated secondary Abs for 1 h, the membranes were analyzed by a gel imaging and analysis system (Champion Chemical, Whittier, CA, USA).

*Limited Trypsin/Proteinase K Digestion*: EVs were incubated with 0.05% trypsin or 25 µg mL^−1^ proteinase K at room temperature to digest surface proteins only. After digestion, 5× loading buffer was added to samples, samples were then incubated at 100 °C for 10 min to stop reaction prior to immunoblots.

*Uptake of EVs*: A total of 5 × 10^5^ spleen cells from OVA‐immunized mice were incubated in 500 µL complete medium with 50 µg PKH26‐labeled OVA‐T‐EVs or WT‐T‐EVs in 24‐well plates for 4 h at 37 °C (5% CO_2_). The cells were collected and washed twice with PBS (with centrifugation at 300 g for 10 min) and then stained with fluorescent conjugated anti‐CD3 and anti‐CD19 mAbs for 30 min at 4 °C. The samples were analyzed using flow cytometry. For CLSM analysis, 3 × 10^5^ isolated B cells (purity > 95%) from OVA‐immunized mice were incubated with 50 µg PKH67‐labeled OVA‐T‐EVs or WT‐T‐EVs for 4 h, washed with PBS and stained with fluorescent conjugated anti‐CD19 mAbs. The cells were then analyzed using a DeltaVision Spectris microscope (Applied Precision, LLC, WA).

*Vaccination and EVs Treatment*: For the isolation of CD4^+^ T cells, BALB/C mice were immunized with 100 µL of 20 µg mL^−1^ HBsAg (i.m.) at day 0 and day14, and the mice were sacrificed on day 16. For EVs treatment, mice were immunized with 100 µL of 20 µg mL^−1^ HBsAg (i.m.) at day 0 and day 14, and 50 µg HB‐T‐EVs/OVA‐T‐EVs/WT‐T‐EVs was intravenously injected (i.v.) at the same time as immunization. Serum, which was used for the analysis of HBsAb IgG, IgG2a, and IgG1, was collected by tail bleeding on days 16, 23, 30, 40, and 50. The mice were sacrificed on day 50, and spleen cells and bone marrow cells were collected and analyzed by flow cytometry.

*Enzyme‐Linked Immunosorbent Assay*: An ELISA was conducted to evaluate the antibody responses in HBsAg‐vaccinated mice by determining the HBsAb IgG, IgG2a, and IgG1 levels. Briefly, 100 µL serum diluted in 2% BSA/PBS (1:200 for IgG2a and IgG1 and 1:1000 for IgG) was added to HBsAg‐precoated 96‐well plates and incubated for 1 h at 37 °C, followed by the addition of HRP‐conjugated anti‐IgG, IgG2a or IgG1 Abs at room temperature for 1 h. Then, 100 µL of TMB was added for 10 min at 37 °C, and the reaction was stopped with H_2_SO_4_. The OD values of the samples were measured at a wavelength of 450 nm using a microplate reader (Bio‐Rad).

*B Cell Response Assay*: B cells were isolated from the spleens of OVA‐immunized mice with anti‐CD19 biotin and antibiotin microbeads. The purity was >95%. A total of 5 × 10^5^ B cells were cultured in 200 µL complete medium (in 96‐well flat bottom plates). The cells were either stimulated with OVA‐T‐EVs or WT‐T‐EVs. Functional grade purified anti‐IgM and CD40 mAb stimulation was used as a positive control, and PBS was used as a negative control. After 48 h, the B cells were collected and incubated with fluorescent conjugated mAbs against CD86, CD80, CD40, and MHCII and then analyzed by flow cytometry. For the B cell proliferation assay, 5 × 10^5^ B cells were labeled at 37 °C with 2.5 × 10^−3^ m CFSE for 10 min. The labeled B cells were washed three times with complete medium (with centrifugation at 300 × *g* for 10 min) and cultured in complete medium in 96‐well flat bottom plates. The B cells were either stimulated with OVA‐T‐EVs or WT‐T‐EVs. After 4 d of culture (37 °C and 5% CO_2_), the cells were collected and analyzed by flow cytometry. Furthermore, the culture supernatant was collected, and total IgG and OVA‐specific IgG antibodies were analyzed by ELISA.

*Construction of the CD40L Knockdown EL‐4 Cell Line*: EL‐4 cells were cultured in complete medium (RPMI 1640 with 10% fetal bovine serum), and the medium was changed one day before transfection. When cells reached 70–80% confluence, CD40L shRNA recombinant plasmids (GenePharma, Shanghai, China) were transfected into the cells using Lipofectamine 2000 transfection reagent (Thermo Fisher Scientific). Positive clones were screened using 500 µg mL^−1^ G418. After abundantly cultivating the positive cells, CD40L interference efficiency was assessed by western blotting.

*Statistical Analysis*: The statistical significance of differences between groups was analyzed by ANOVA and Student's *t*‐test using Prism version 6.02 (GraphPad Software, Inc., San Diego, CA, USA). The data are presented as the mean ± SEM from at least three independent experiments. *P*‐values ≤ 0.05 were considered statistically significant.

## Conflict of Interest

The authors declare no conflict of interest.

## Supporting information

Supplementary
