# Supplementary material for: CD4+ T Cell‐Released Extracellular Vesicles Potentiate the Efficacy of the HBsAg Vaccine by Enhancing B Cell Responses
Source: Adv Sci (Weinh). 2019 Sep 30;6(23):1802219. doi: 10.1002/advs.201802219 (PMC6891927; doi:10.1002/advs.201802219)
Supplement: Supplementary file 1 — Supplementary [file ADVS-6-1802219-s001.pdf]

Copyright WILEY-VCH Verlag GmbH & Co. KGaA, 69469 Weinheim, Germany, 2019.

ADVANCED SCIENCE
Open Access

Supporting Information

for *Adv. Sci.,* DOI: 10.1002/advs.201802219

CD4^{+} T Cell-Released Extracellular Vesicles Potentiate the Efficacy of the HBsAg Vaccine by Enhancing B Cell Responses

Jian Lu, Jing Wu, Feiting Xie, Jie Tian, Xinyi Tang, Hongye Guo, Jie Ma, Ping Xu,* Lingxiang Mao,* Huaxi Xu, and Shengjun Wang**

Supplementary Figures

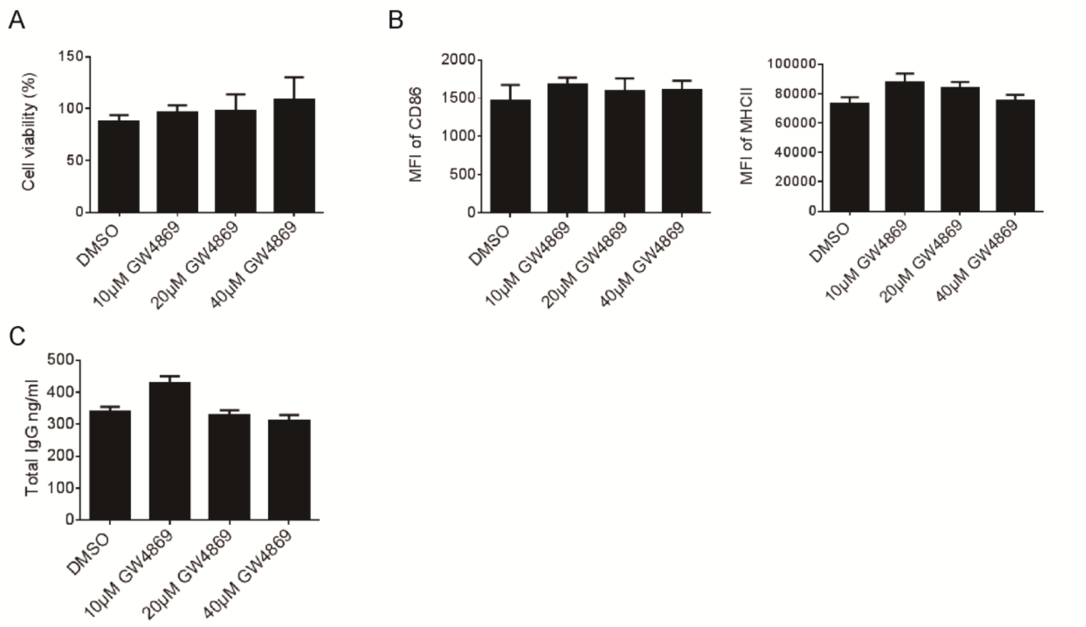

Supplementary Figure 1: The effects of GW4869 on $CD4^+$ T cell viability and B cell responses. (A) CCK-8 assays were performed to investigate the survival of $CD4^+$ T cells treated with different concentrations of GW4869. (B) Flow cytometry analysis of CD86 and MHC Ⅱ expression on the surface of B cells treated with DMSO or GW4869. (C) Total IgG level in supernatant of B cells treated with DMSO or GW4869 was analyzed by ELISA. The data are from three independent experiments (mean and s.e.m.).

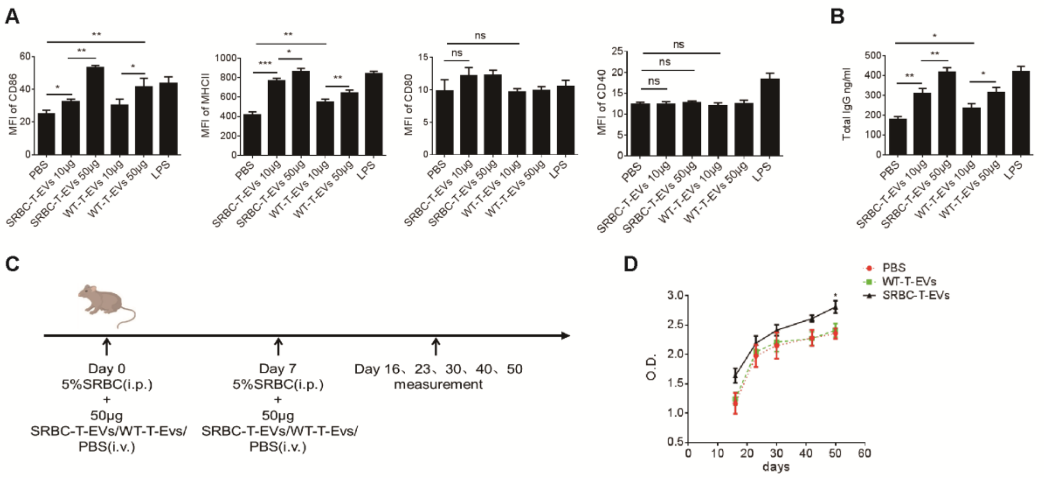

Supplementary Figure 2: CD4+ T cell EVs promote B cell responses and enhance anti-SRBC IgG production in BALB/C mice immunized with SRBC. (A) A total of 5×10^5 B cells isolated from SRBC-immunized mice were incubated with different doses of CD4+ T cell EVs derived from SRBC-immunized mice or wild-type mice (termed SRBC-T-EVs and WT-T-EVs, respectively) for 48 h. The activation of B cells was evaluated by the expression of CD86, CD80, CD40 and MHC II. (B) For the antibody production experiment, 5×10^5 B cells were incubated with SRBC-T-EVs or WT-T-EVs for 4 d; the culture supernatant was collected, and the total IgG was analyzed by ELISA. (C) A schematic of the in vivo experiment. BALB/C mice were immunized with SRBC (i.p.) together with SRBC-T-EVs/WT-T-EVs or PBS treatment (i.v.), and serum was collected on days 16, 23, 30, 40 and 50. (D) The absorbance of anti-SRBC IgG was quantified by ELISA (at a wavelength of 450 nm). *$P<0.05$ and **$P<0.01$ (Student's t-test). The data are from two independent experiments with five mice per group (A, B, and D; mean and s.e.m.).

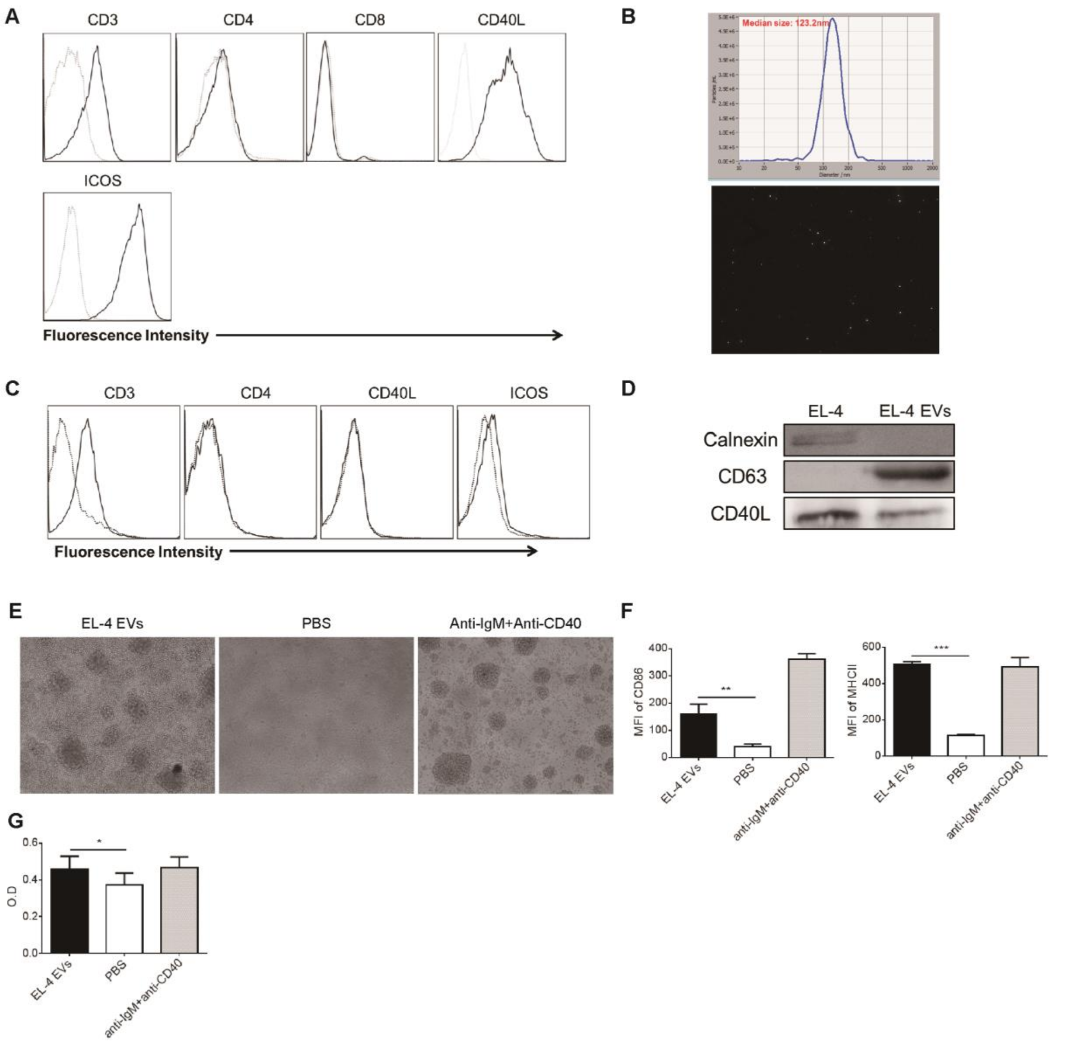

Supplementary Figure 3: Phenotypic characterization of EL-4 cells and analysis of their EVs function. (A) A total of 1×10^6 EL-4 cells were stained with a panel of mAbs (solid lines) or isotype-matched mAbs (dotted lines) and then analyzed by FCM. (B) A representative chart of the size distribution of EL-4 EVs analyzed by Zeta View (a nanoparticle tracking analysis device; Particle Matrix, Germany). (C) The phenotypes of EL-4 EVs were analyzed by FCM. (D) Calnexin, CD63 and CD40L expression of EL-4 EVs were analyzed by western blotting. A total of 5×10^5 B cells isolated from wild-type mice were incubated with 50 μg EL-4 EVs or functional grade purified anti-mouse IgM and CD40 or PBS for 48 h. The proliferation of B cells was evaluated by microscopy (E), and the expression of CD86 and MHC II was analyzed by FCM (F). The total IgG in the culture supernatant was analyzed by ELISA (G). *$P<0.05$, **$P<0.01$ and ***$P<0.001$ (Student's t-test). The data are representative of three independent experiments (A, B, C, D, and E) or are from three independent experiments (F and G, mean and s.e.m.).

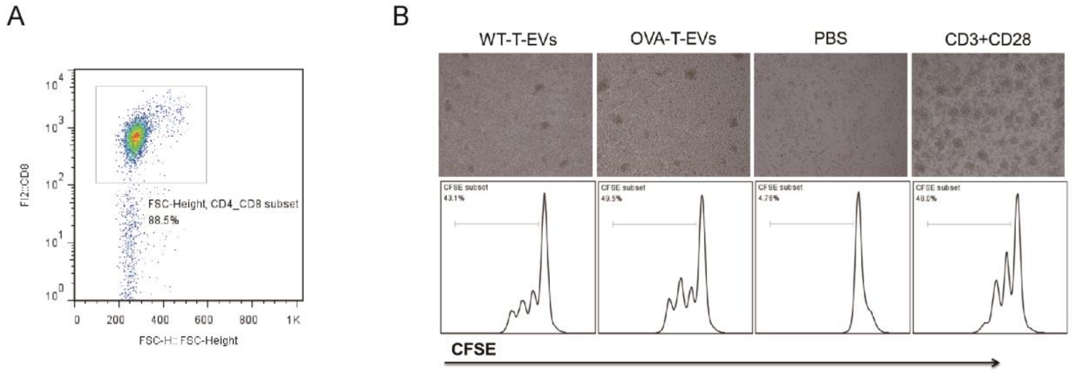

Supplementary Figure 4: CD4$^+$ T cell EVs promote the proliferation of CD8$^+$ T cells in vitro. (A) The purity of CD8$^+$ T cells isolated from OVA-immunized mice was analyzed by flow cytometry. (B) A total of 5×10^5 CFSE-labeled CD8$^+$ T cells were incubated with 50 μg OVA-T-EVs or WT-T-EVs for 4 d, and the proliferation of CD8$^+$ T cells was analyzed by microscopy and flow cytometry. The data are from two independent experiments.
